# A Single-Centre Review of Outcomes of Delayed Admission to a Burns Unit

**DOI:** 10.3390/ebj7020032

**Published:** 2026-06-10

**Authors:** Quentin Isaacs, Chrysis Sofianos, Adelin Muganza, Brian Brummer

**Affiliations:** 1 Division of Plastic Surgery, Department of Surgery, School of Clinical Medicine, Faculty of Health Sciences, University of the Witwatersrand, Johannesburg 2050, South Africa; sofianosc@gmail.com; 2 Division of General Surgery, Department of Surgery, School of Clinical Medicine, Faculty of Health Sciences, University of the Witwatersrand, Johannesburg 2050, South Africa; amuganza@gmail.com; 3 National Health Laboratory Service, Division of Public Health Surveillance and Response, National Institute for Communicable Diseases, Johannesburg 0001, South Africa; studystatsbb@gmail.com

**Keywords:** delayed admissions, burns, South Africa, length of stay, resource-limited settings, retrospective audit

## Abstract

**Background:** Timely admission to a specialised burn unit is considered crucial for optimising outcomes in burn patients. However, the impact of delayed admission on hospital length of stay and clinical outcomes remains unclear, particularly in resource-constrained settings such as South Africa. This retrospective study aimed to determine whether admission to a burn unit more than 24 h after injury was associated with increased length of stay, sepsis, or mortality. **Methods:** A retrospective case-audit study was conducted at the Chris Hani Baragwanath Academic Hospital Adult Burn Unit, Johannesburg, from January 2018 to December 2022. Patients were categorised into early (≤24 h) and delayed (>24 h) admission groups. The primary outcome was length of stay; secondary outcomes included sepsis incidence and in-hospital mortality. **Results:** A total of 123 files were analysed; 71 (58%) were admitted within 24 h. The median length of stay was 14 days, with no statistical difference between the two groups (*p* = 0.7). The overall mortality rate was 13%, with 68% occurring in the early admission group. Sepsis occurred in 27% of patients. Multivariate analysis revealed that early admission was independently associated with longer length of stay. **Conclusions:** In this single-centre retrospective case note audit with a limited sample size and significant risk of selection bias, delayed admission to a burn unit was not associated with increased length of stay, mortality, or sepsis. However, these findings should be considered preliminary and require confirmation in larger, prospective studies. The higher rate of surgical intervention in the delayed admission group warrants further investigation.

## 1. Introduction

### 1.1. Epidemiology and Local Context

Developing nations account for the majority of the global burn burden [1]. In recent years, there has been a focused effort to develop global surgery initiatives and improve surgical outcomes. The burden of burns in South Africa amounts to 1.6 million cases per year, and research suggests there are 8140 admissions to burn centres across the country annually, with 2600 in Gauteng province alone [1]. There were 54,160 deaths due to trauma as per national statistics published in 2018, with burns accounting for 7.2% of these cases, which is lower than South African burn units’ overall mortality rate of 13.21% [2,3]. Findings in this study may be skewed due to a predominantly paediatric population.

Chris Hani Baragwanath Academic Hospital (CHBAH) is a 3400-bed tertiary referral centre located in Soweto, Johannesburg. It serves a catchment population of approximately 7 million people across Gauteng and surrounding provinces. The hospital functions as a high-volume Level 1 trauma centre and receives referrals from numerous district and regional hospitals across a wide geographical area. The Adult Burn Unit has 22 dedicated beds and admits approximately 280–300 adult patients annually. The unit admits patients according to American Burn Association (ABA) referral criteria, including partial (>10% TBSA) and full-thickness burns, burns involving critical areas (face, hands, feet, perineum), electrical and chemical burns, and burns with concomitant inhalation injury. The hospital also has a separate paediatric burn unit; this study focused exclusively on adult patient admissions. If severe burns are not accommodated in the burn unit due to bed capacity, the trauma unit admits them and takes over their management. The unit has one full-time burn surgeon, one plastic surgery registrar and junior doctors who rotate through the unit.

### 1.2. Cost of Care

South African burn surgeons have reported that the inpatient cost of managing scald burns occupying 20% of the patient’s total body surface area (TBSA) amounts to ZAR 22,394 (approximately USD 1200) for the inpatient phase of care, almost tripling with TBSA of 21–30%, and increasing exponentially if over 30% [4]. These costs encompass the entire inpatient episode from admission to discharge, including surgical procedures, dressings, intensive care, and allied health services. They also showed that the mechanism of injury plays a role in the cost of managing paediatric burns—flame burns incur much higher costs than scalds due to longer admissions and greater surgical requirements [4,5]. Understanding factors that influence length of stay (LOS) is therefore critical for resource allocation and cost containment in a publicly funded healthcare system.

### 1.3. Surgical Timing and Length of Stay

As burn care has evolved over the decades, metrics for monitoring outcomes have changed. Since the adoption and advocacy of early excision and grafting to improve outcomes and reduce mortality, research has focused on LOS as a marker of centre performance [6,7]. This continuous quantitative variable helps indicate the quality of care and complication rates, as increased complications are directly correlated with increased LOS [6,7].

At CHBAH, early excision and grafting is the standard of care for deep partial- and full-thickness burns. However, several factors contribute to delays in surgical intervention: limited dedicated burn theatre time (the unit has access to one theatre inside the unit which is shared between the adult and paediatric burn units), after hours competing with trauma surgery priorities and all other surgical emergencies, logistical challenges of transferring patients from the burn unit to the main theatre complex, and occasional delays in patient transfer from referring facilities. These resource constraints are typical of many public sector hospitals in developing nations and provide important context for interpreting surgical timing and LOS outcomes.

While we have no control over burn patients’ sex, age, TBSA or presence of inhalation injury—which all contribute to LOS and mortality—other potentially modifiable aspects must be assessed to improve outcomes [6,7,8,9,10].

### 1.4. Delayed Transfer and Admission to a Burn Unit

Providing burn care is costly. Various studies have assessed the measures within our control that can be improved [4,10,11]. These studies highlighted delayed admission, defined as a wait of greater than 24 h to enter a burn centre, and associated factors including transfer distance, referring hospital capacity, ICU bed availability, and need for stabilisation before transfer [8,11,12,13,14]. If these measures can be improved, resulting in shorter stays and better outcomes, it will decrease the cost of managing these patients. Large burns require specialised care, and any delay in that care can be detrimental.

The benefits of early resuscitation and adequate first aid before burn centre admission are well established. Patients who receive appropriate fluid resuscitation, wound care, and airway management at referring hospitals may be better protected against the negative effects of transfer delays. Conversely, inadequate initial management may compound the risks of delayed admission. In our local context, decisions about whether to transfer patients with extremely severe burns (e.g., >60% TBSA in elderly patients or those with poor prognosis) involve multidisciplinary discussion and consideration of resource availability, likelihood of meaningful recovery, and patient/family wishes. Such patients may not be transferred to the burn unit if the anticipated outcome is deemed futile.

Research has shown that delayed management at a Level 1 trauma centre can adversely affect trauma patients, particularly burn patients requiring intubation [15,16]. It has been reported that a transfer delay to a burn centre of more than 24 h resulted in poorer outcomes, including higher rates of sepsis, organ dysfunction, and longer hospital stays [16]. Note that this particular study involved paediatric burn patients.

Studies focused on mortality have noted that age, gender, TBSA, inhalation injury, delay to intubation of more than 16 h after time of burn, intubation during transfer, need for ventilatory support and direct admission to a burn unit were associated with increased risk of mortality [5,6,8,10,12,13,15,17]. Further scrutiny of these international studies showed that patients classified as having delayed admissions were often admitted within 24 h due to very efficient prehospital transfer systems, while those with delays beyond 24 h frequently received adequate management at referring hospitals. The higher mortality in direct admissions may have been due to the severe extent of the burns, as severely burned patients with poor prognoses might have been transferred directly to burn centres without initial stabilisation elsewhere [8,10,13,15,17].

In developing countries, patients present an average of five days from the date of injury, predominantly exhibiting dehydration, wound infection, systemic sepsis and acute kidney injury, with an overall mortality rate of 11.4% [5]. The need for blood transfusion, excision and grafting, and the number of procedures performed are independent factors that contribute to LOS [18]. Unfortunately, mortality and time from burn to admission were not among the outcomes measured in this study. Other research has contradicted earlier findings and found no statistically significant association between mortality, injury severity and length of stay in patients transferred directly from other facilities [8,15].

### 1.5. Objective

This study aimed to evaluate whether a delay of greater than 24 h from burn injury to admission to a burn unit was associated with increased LOS, sepsis, or mortality in a resource-constrained South African setting.

## 2. Materials and Methods

### 2.1. Study Design and Setting

This retrospective case-audit study was designed to evaluate whether a delay of more than 24 h from burn injury to admission increased LOS in a single-centre burn unit, namely, the CHBAH Adult Burn Unit in Johannesburg, South Africa. This 22-bed unit serves a large urban and peri-urban population and functions as a tertiary referral centre for the region, admitting approximately 280–300 adult patients annually.

### 2.2. Study Population

The study population comprised all patients admitted to the burn unit from 1 January 2018 to 31 December 2022. Requirements for admission were in accordance with the American Burn Association (ABA) guidelines: partial thickness burns > 10% TBSA, full thickness burns, burns involving critical areas (face, hands, feet, perineum), electrical and chemical burns, burns with inhalation injury, and burns in patients with comorbidities that could complicate management. All patients admitted to the unit were eligible for inclusion, including those with superficial burns requiring admission for pain management or social reasons. Patients were identified using admission records; hard copies of these documents were collected from the hospital records department. No exclusion criteria were applied during the initial identification phase to ensure a representative sample and minimise bias.

We obtained permission to conduct the research from the Medical Advisory Committee and the CEO of Chris Hani Baragwanath Academic Hospital. Ethical approval was obtained from the University of the Witwatersrand Human Research Ethics Committee (Medical) (HREC), clearance certificate M2300127 on 22 August 2023.

A total of 1410 patients were admitted over the five years. Based on this population, a sample size of 336 patients would have been required for adequate statistical power (confidence level 95%, margin of error 5%, expected proportion 50%). File retrieval was dependent on the records department staff. Records being stored between the main hospital and the St John’s Eye Hospital satellite storage facility contributed to poor file retrieval. The records department staff provided files based on availability; the research team did not select specific files. Patients were excluded from analysis if their files were incomplete or missing critical data (time of burn, admission date, LOS, or outcome).

### 2.3. Data Collection

A single investigator (Q.I.) collected all the data using a standardised data collection sheet created to record all relevant information from the patient files. Data collected included patient demographics (sex, age and comorbidities), clinical burn data (time of burn, mechanism, TBSA, length of stay, sepsis, surgery and death). The primary investigator was not involved in the clinical care of any patients included in this audit to reduce bias. Data collection followed a predefined protocol, and any uncertainties in data extraction were discussed with senior investigators (C.S., A.M.) to reach consensus. The patients were allocated study numbers, and all identifiers were removed to ensure patient confidentiality. The data were transcribed into Microsoft Excel 365; the hard copies were stored in a locked cupboard and accessed only by the primary investigator. The database was stored on the principal investigator’s password-protected laptop and was accessible only to the investigators. Due to the limited number of files, only one data collector was used, and the burn surgeons did not perform data collection.

For this study, delayed admission was defined as presentation to the burn unit more than 24 h after the time of injury, consistent with previous literature [11,12]. Time of injury was obtained from patient records or referral letters; when discrepancies existed, the earliest documented time was used. Surgical procedures included debridement, skin grafting, and escharotomies. Escharotomies were recorded as surgical procedures when performed in the operating theatre; bedside escharotomies were not consistently documented and could not be reliably analysed.

### 2.4. Data Analysis

Statistical analysis was completed by a co-investigator with epidemiological expertise (B.B.). The data were transferred from Microsoft Excel 365 into R (version 4.5) for statistical analysis. Observations with extreme values that were clinically implausible (e.g., time to admission >100 days without clinical justification) were reviewed individually. In the local context, transfers occurring more than 100 days post-injury would be highly unusual and likely reflect either data-capture errors or exceptional circumstances (e.g., patients with complications initially managed elsewhere). Those files were subsequently retrieved to ensure no data-collection errors. If cases could not be verified, they were excluded from analysis to prevent data entry errors from skewing results. This conservative approach was taken to ensure data integrity, although we acknowledge that some genuine outliers may have been excluded.

We describe the data using medians and interquartile ranges (IQRs) for continuous variables and counts and percentages for categorical variables. The Wilcoxon rank-sum test was used to compare continuous variables between groups, and the chi-square test was used for categorical variables. Fisher’s exact test was used where expected cell counts were less than 5.

We conducted both univariate and multivariable linear regression to assess the association between time to admission and LOS. Time to admission was analysed both as a binary variable (early vs. late) and a continuous variable to explore potential non-linear relationships. Variables included in the multivariable model were selected based on clinical relevance and prior literature, including age, TBSA, inhalational injury, sepsis, and mortality status. We have reported modified Baux and Lethal Area 50 (LA50) scores. The modified Baux score was calculated as TBSA + age (+17 if there was an inhalation injury), and LA50 was determined as the proportion of patients who died in each TBSA category (in 10% increments).

Due to collinearity between the Baux score and its components (age, TBSA, inhalation injury), the Baux score was included only in univariate analysis and excluded from the multivariable model to avoid multicollinearity.

A *p*-value < 0.05 was considered statistically significant.

## 3. Results

A total of 134 patient folders were located from the records department. After excluding 11 incomplete files (missing critical data such as time of injury, admission date, or outcome), 123 patients were included in the final analysis. This represents only 8.7% of the total 1410 patients admitted during the study period, highlighting a significant risk of selection bias.

### 3.1. Demographics

Table 1 illustrates a comprehensive breakdown of the results and the patient demographics. The patients were mostly male (64%) and had a median age of 34 years (IQR 26–42). The early admission group had a median age of 32 years (IQR 25-40) and the late admission group had on of 36 years (IQR 27–44) (*p* = 0.26). A total of 71 patients (58%) were admitted within 24 h of their burn injury, and 52 (42%) were admitted after more than 24 h. Among the latter group, the median time to admission was 4 days (IQR 2–7 days) (*p* < 0.001 compared to the early group).

HIV was the most common comorbidity in the study population, documented in 18% of patients (this variable was not included in the final table due to inconsistent documentation; HIV status was recorded in 22 patients, with 18% positivity among those tested). Other comorbidities included hypertension (9.8%), right ventricular dysfunction (18%), diabetes mellitus (4.9%), and epilepsy (4.1%).

Flame burns (47%) and hot water/scalding (29%) were the most frequent burn mechanisms, followed by electrical burns (17%). Among patients with electrical burns, 86% (18/21) were in the early admission group, reflecting the recognised severity of electrical injuries and protocols mandating rapid transfer. Among patients with flame burns, 59% (34/58) were in the early admission group.

The median TBSA in the study population was 17% (IQR 10–27%), with early admission at 16% (IQR 10–25%) and late admission at 18% (IQR 13–29%). At our institution, burn physicians do not routinely attend the emergency department for initial assessment; emergency medicine physicians initiate TBSA estimation and fluid resuscitation. The emergency department TBSA assessment showed a strong correlation with the final burn unit assessment (Spearman’s correlation rho = 0.94). However, the emergency department overestimated the proportion of patients with TBSA > 50% by 11% (95% confidence interval [CI] 5–16%). A total of 43 patients (34%) suffered a TBSA of greater than 20%. Twenty-three patients had inhalational injuries, and the early admission group comprised the vast majority of these patients at 78% (*p* = 0.027; Table 1).

Of the early admission group, 70% (n = 50) were admitted via the trauma emergency unit compared to only 25% (n = 13) of the late admission group. The late admission group had an additional 35% (n = 18) transferred from the general trauma ward to the burn unit within the same hospital, indicating that these patients were already in the hospital but awaiting admission to the burn unit due to bed shortages. The remaining late admissions were referred from regional hospitals (n = 15, 29%) or presented directly from home (n = 6, 11%).

### 3.2. Primary Outcome: Length of Stay

The median LOS was 14 days (IQR 8–25) across the entire study population, and this did not differ significantly between those who experienced early or late admission (*p* = 0.74). There was no difference in median Baux scores between the early and late admission groups. The Baux score for those who died was 88 (IQR 81–101) compared to 53 (IQR 42–69) for survivors (*p* < 0.001).

**Figure 1:**
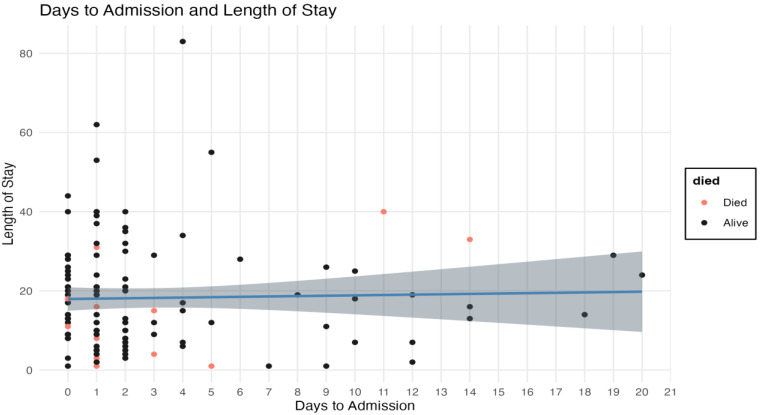
Scatterplot and linear regression with confidence intervals of length of stay in days (y) by days to admission (x), with separate panels for early (≤24 h) and late (>24 h) admission groups.

As shown in Figure 1, there was no linear correlation between days to admission and length of stay when time to admission was analysed as a continuous variable. Figure 1 presents separate scatterplots for early and late admission groups with regression lines, demonstrating no significant relationship in either group. Sensitivity analysis using categorical time-to-admission groups (<24 h, 1–3 days, >3 days) also revealed no significant differences in LOS between groups.

Reasons for delayed admission to the unit included awaiting COVID-19 results (18%, n = 22), awaiting an ICU bed (15%, n = 19) and delayed presentation (4%, n = 5). No reason was stipulated in the files for most patients (63%). Within the late admission group specifically, 23% had delays due to pending ICU beds, 7% due to pending COVID-19 results, 5% due to delayed presentation, and the remaining 65% had no documented reason.

In univariate analysis, a need for surgery was associated with increased LOS (Beta 9.2, 95% CI: 3.9–14; *p* < 0.001), particularly a need for debridement and skin grafting. An increased Baux score was associated with increased LOS: for each unit increase in Baux score, LOS increased by 0.15 days (95% CI: 0.05–0.26; *p* = 0.005).

Under univariate conditions, significant predictors of LOS included age (*p* < 0.001), TBSA (*p* = 0.002), need for surgery (*p* < 0.001) and sepsis (*p* < 0.001). When controlling for other factors in multivariable analysis, only age, TBSA (borderline significance), death, and early admission remained associated with LOS (Table 2). Notably, in the multivariable model, early admission was independently associated with longer LOS (Beta −5.8 for late admission, meaning early admission patients stayed approximately 5.8 days longer on average when other factors were held constant; 95% CI: −11 to −0.80; *p* = 0.024).

### 3.3. Secondary Outcomes: Sepsis and Mortality

The sepsis rate in the study population was 27% (n = 33), with no difference between the early and late admission groups (27% vs. 27%, *p* = 0.98). However, among patients who developed sepsis, those in the early admission group had a higher proportion of flame and electrical burns, which are typically associated with greater tissue destruction and infection risk.

The overall mortality rate was 13% (n = 16), and 69% (n = 11) of the deaths occurred in the early admission group. The median age of patients who died was 45 years (IQR 34–69) compared to 33 years (IQR 25–39) for those who survived (*p* = 0.004). Males made up 69% (n = 11) of those who died. Only 25% (n = 4) of patients with inhalation injuries died (*p* = 0.5), suggesting that timely recognition and management of airway compromise may have been effective. Of the patients who died, flame burns accounted for 88% (n = 14), and hot water burns accounted for the remaining two deaths (*p* < 0.001). No electrical burn patients died.

The vast majority of deaths occurred in 2020 (n = 15, 94% of all deaths), coinciding with the peak of the COVID-19 pandemic. During this period, our unit, like many globally, observed increased pressures on ICU bed availability, staffing shortages, and potentially delayed presentations. Additionally, there is some evidence that self-harm presentations increased during lockdown periods, although this was not specifically documented in our mortality cohort. Only 2 patients were parasuicide/self-harm cases. Notably, 94% of the patients who died developed Gram-negative sepsis (predominantly Pseudomonas and Klebsiella species). The median TBSA in patients who died was 37% (IQR 34–50%) compared to 15% (IQR 10–22%) in survivors—a statistically significant difference (*p* < 0.001). The LA50 for the study population was 30–40% TBSA.

Table 3 presents an overview of study characteristics related to mortality. A total of 41 patients (33% of the cohort) required surgery, with late admissions comprising 63% (n = 26) of this group. Among those requiring surgery, debridement was required by 33% (n = 17) in the late admission group compared to 12.5% (n = 9) in the early admission group, while 18 patients in the late group required skin grafts compared to 8 in the early group. This higher rate of surgical intervention in the delayed-admission group (50% vs. 21%, *p* < 0.001) is a key finding that may reflect progression of burn wound depth during the waiting period or selection of patients with initially indeterminate-depth burns that ultimately required surgery. Escharotomies were performed in 8 patients (6 early, 2 late) and were included in the surgical count.

### 3.4. Summary of Multivariate Analysis

Multivariable analysis showed that LOS was independently associated with age, TBSA (borderline significance), death, and early admission. The association between early admission and longer LOS (Beta −5.8 for late admission) persisted after controlling for injury severity, suggesting that this finding is not simply due to confounding by indication. However, residual confounding cannot be excluded.

Multivariate analysis with respect to deaths identified factors associated with increased mortality as age, TBSA, flame burns, and Gram-negative sepsis, particularly Pseudomonas and Klebsiella species. Sepsis was associated with increased LOS only under univariate conditions, suggesting that the factors predisposing to sepsis (large TBSA, flame burns) are themselves the primary drivers of prolonged hospitalisation.

## 4. Discussion

This single-centre retrospective case-audit study from Johannesburg, South Africa, found that delayed admission (>24 h) to a specialised burn unit was not associated with increased LOS, sepsis, or mortality. In fact, multivariate analysis revealed that early admission was independently associated with longer LOS. This finding likely reflects the effectiveness of current triage systems in rapidly identifying and transferring the most severely injured patients. However, these findings must be interpreted with considerable caution due to the study’s significant limitations, particularly the small sample size (8.7% of the total eligible population) and high risk of selection bias.

### 4.1. Demographic and Clinical Context in Comparison with Other Centres

The study population had a similar distribution of demographic characteristics to those studies conducted in both developing and developed centres. Outcomes in a South African cohort with a similar age and sex distribution have been reported previously [3]. The median age of 34 years and male predominance (64%) align with findings from Queensland, Australia (median age 37 years, 67% male) and the Western Cape, South Africa (68% male) [8,19]. This consistent demographic pattern reflects the greater occupational and risk-taking exposure among young adult males across diverse settings.

The overall mechanisms of injury were similar to those of other South African studies. However, we observed a higher proportion of electrical burns (17%) than in developed nations, where electrical burns typically constitute <5% of admissions [3,8,10,11,19]. This likely reflects differences in occupational safety standards, informal housing with unsafe electrical installations, and the high prevalence of electrical infrastructure theft and illegal connections in South African townships. The predominance of flame burns (47%) is consistent with other low- and middle-income country (LMIC) settings where open-flame cooking and heating are common [5].

A crucial distinction between our cohort and those from high-income countries is burn severity. In an Australian and New Zealand Burn Association (ANZBA) registry study, fewer than 10% of patients had TBSA > 20% [10]. In contrast, 34% of our patients had TBSA > 20%, and the median TBSA of 17% is substantially higher than the ANZBA median of approximately 5–8% [8,10]. This disparity underscores the greater severity of burn injuries managed in developing nations and highlights the importance of context-specific research. It is worth noting that, although we follow ABA transfer guidelines, ANZBA uses different referral criteria, which may result in the transfer of patients with smaller burns, contributing to differences in case mix between studies.

### 4.2. Length of Stay and Admission Timing: Comparison with Published Literature

Our primary finding—that delayed admission was not associated with prolonged LOS—contrasts with older literature, such as a 1999 Boston study that included 78 paediatric patients, which found that transfer delay >24 h resulted in significantly longer LOS (mean 32 vs. 18 days, *p* < 0.05) and higher complication rates [16]. However, this cohort had a mean TBSA of 42% in the delayed group, considerably larger than our delayed group’s median of 18%, and the study was conducted over two decades ago, when burn care protocols differed substantially.

Our results align with more recent studies from developed and developing settings. No association was found between transfer status and either LOS or mortality after adjusting for burn severity in a study on 600 patients in Queensland [8]. A study on 2951 patients from the ANZBA registry reported that transfer time > 24 h was not associated with increased mortality; in fact, transferred patients had lower unadjusted mortality, which disappeared after adjustment for severity [10]. In a United States study, no difference in outcomes between transferred and directly admitted patients was found after controlling for injury characteristics [13].

In the LMIC context, a study including 280 burn patients in rural Tanzania found that, while delayed presentation (median 5 days) was associated with higher rates of wound infection and sepsis at admission, it did not independently predict mortality after controlling for TBSA and age [5]. A report from China stated that delayed hospital admission did not increase sepsis risk when patients received adequate prehospital care, suggesting that the quality of interim management may be more important than transfer speed per se [14]. Another explanation for why delayed admission produced no statistical difference in LOS could be that the early admission group had a higher rate of sepsis, possibly due to a higher percentage of flame and electrical burns. These burns are typically more extensive, placing victims at a higher risk of sepsis [20,21].

The multivariate analysis finding that early admission was independently associated with longer LOS requires careful interpretation. This paradoxical finding does not imply that delaying admission is beneficial. Rather, it most likely indicates that patients with the most severe injuries—those who will inevitably require prolonged hospitalisation—are appropriately prioritised for rapid transfer to the burn unit. This interpretation is supported by the early admission group having higher rates of inhalational injury (25% vs. 9.6%, *p* = 0.027), more flame burns, and a greater proportion of electrical injuries. Other studies have reported similar findings, noting that patients admitted early to burn units often have increased LOS and higher mortality due to greater burn severity [8,10].

The lack of association between inhalation injury and LOS in our multivariate model, despite its significance in univariate analysis, suggests that other factors (age, TBSA, need for surgery) are more powerful drivers of hospitalisation duration. This aligns with a systematic review of 23 studies, which identified age, TBSA, and need for surgery as the most consistent predictors of LOS across multiple studies. At the same time, inhalational injury showed inconsistent associations after multivariable adjustment [7]. 

### 4.3. Surgical Intervention in Delayed Admissions

One of the most striking findings in our study was the significantly higher rate of surgical intervention in the delayed-admission group (50% vs. 21%, *p* < 0.001). This finding has several potential explanations and warrants comparison with existing literature.

First, patients with delayed presentation may have sustained deeper burns than initially appreciated, or their wounds may have progressed during the waiting period, converting partial-thickness injuries into full-thickness wounds requiring excision and grafting. A study of a paediatric cohort that delayed wound excision (>7 days) found increased blood loss and longer hospital stays [21]. However, this study focused on the timing of surgery rather than admission.

Second, the unit’s policy of using honey-based dressings (Melladerm™) for deep partial and intermediate-depth burns to allow demarcation before surgery may result in a watchful-waiting period that inherently delays surgical intervention. This approach is supported by the literature, which suggests that delayed grafting of indeterminate-depth burns may reduce the extent of excision required [20].

Third, resource constraints—including limited dedicated burn theatre time (2–3 days per week), competing trauma surgery priorities, and the logistical challenges of performing after-hours debridement in a unit separate from the main theatre complex—may contribute to delayed first excisions, particularly for patients already waiting for admission. These challenges are well-described in resource-limited settings and represent an important area for quality improvement [4,5].

A Lebanese study of 352 patients found that the number of surgical procedures was an independent predictor of LOS, with each additional procedure increasing LOS by approximately 5 days [18]. Our univariate analysis similarly showed that the need for surgery was associated with a 9.2-day longer hospital stay. While delayed admission itself was not directly associated with longer LOS in our study, the downstream effect of increased surgical requirements among delayed patients may indirectly affect patient morbidity and the use of hospital resources.

The authors have planned a more extensive review of the current cohort of patients who underwent surgery.

### 4.4. Mortality and Severity of Injury

The overall mortality rate of 13% in our study is comparable to the 11.4% reported in Tanzania and the 12.5% reported in another South African centre [3,5]. These rates are substantially higher than those reported in high-income countries: 3.4% mortality in the ANZBA registry, and 2.8% in Queensland [8,10]. This disparity reflects the greater burn severity and resource constraints typical of LMIC settings.

The higher proportion of deaths in the early admission group (69% of all deaths) again reflects the effective triage of severely burned patients to the burn unit. The median TBSA of patients who died was more than double that of survivors (37% vs. 15%, *p* < 0.001), and the LA50 of 30–40% TBSA is consistent with other resource-limited settings where critical care resources may be constrained. In comparison, an LA50 of 40–50% was reported in a US regional burn centre, and an LA50 of 30-40% in Tanzania, similar to our findings [5,17].

The strong association between Gram-negative sepsis (particularly Pseudomonas and Klebsiella) and mortality in our study (94% of deaths had documented Gram-negative infection) underscores the importance of infection prevention and control measures in burn care. Gram-negative sepsis produces more severe systemic illness and deeper wound penetration, necessitating multiple debridements and blood transfusions, which further worsen outcomes [20]. It has been reported that each episode of sepsis increases mortality risk by 20–30% in burn patients, reinforcing the critical need for rigorous infection control protocols [9].

The clustering of deaths in 2020 (94% of all deaths) likely reflects the dual burden of the COVID-19 pandemic on healthcare delivery. During this period, our ICU bed capacity was reduced due to repurposing for COVID-19 patients, staffing was stretched, and there may have been delays in presentation due to lockdown restrictions and fear of hospital attendance. Globally, burn units reported similar challenges; a multi-centre study could help elucidate the pandemic’s true impact on burn outcomes. After this publication, we plan a more detailed look at the COVID period in our study population.

### 4.5. Comparison with International Literature: Synthesis

Our findings contribute to a growing body of literature suggesting that the relationship between transfer timing and burn outcomes is more complex than previously thought. A study of 1087 patients in Seattle found no difference in mortality between transferred and directly admitted patients after adjusting for injury severity [17]. Similarly, a study of 1156 patients in British Columbia reported that direct transport to burn centres did not improve outcomes when severity was accounted for [15]. More recently, researchers in Los Angeles identified factors associated with delayed admission (distance, referring hospital type, weekend presentation) but did not find a direct mortality effect [12].

The key distinction between our findings and older literature may lie in the quality of care at referring institutions. In our context, the majority of delayed admissions came from within the same hospital (trauma unit) or from other surgical training centres, where staff have adequate knowledge of emergency burn care, including fluid resuscitation, wound management, and recognition of inhalation injury requiring intubation. The importance of adequate prehospital and referring hospital care is supported by a Chinese study showing that proper prehospital treatment could mitigate the effects of delayed admission [14]. This contrasts with settings where referring hospitals have limited capacity to provide even basic burn management.

The differences in referral criteria between the ABA guidelines (which we follow) and the ANZBA guidelines (used in comparator studies) may also contribute to variability in findings. ANZBA criteria include smaller burns (>5% TBSA in children, >10% in adults) and lower thresholds for transfer, potentially leading to the transfer of patients with less severe injuries who are better able to tolerate delays. This should be considered when comparing outcomes across studies.

### 4.6. Strengths and Limitations

Strengths: This study addresses an important clinical question in a resource-constrained setting where burn injury burden is high and evidence is limited. We collected comprehensive clinical data and employed appropriate statistical methods, including multivariable adjustment for potential confounders. The inclusion of Baux and LA50 scores facilitates comparison with other populations. The primary investigator was not involved in clinical care, reducing potential bias in data extraction.

Limitations: The study’s limitations are substantial and must be acknowledged when interpreting results.

Selection bias: The most critical limitation is the extremely low file retrieval rate (8.7% of the total eligible population). It is highly plausible that patients with poor outcomes or complex, lengthy admissions had thicker files that were more likely to be in use, off-site, or otherwise unavailable during data collection. Conversely, patients with uncomplicated, short admissions may have had thinner files that were easier to retrieve. If either scenario occurred, our sample would not be representative of the overall burn unit population, and our conclusions could be substantially biased. The lack of statistically significant differences between groups may reflect a Type II error (false negative) due to insufficient power rather than a true absence of effect.

Information bias: Retrospective data collection relies on the accuracy and completeness of medical records. The high proportion of “Not Stated” reasons for delayed admission (63%) and missing comorbidity data (e.g., HIV status incompletely recorded) introduces information bias. TBSA estimation by emergency physicians, while strongly correlated with burn unit assessment, showed systematic overestimation of very large burns, which could affect severity adjustment.

Single-centre design: Findings from a single tertiary centre in urban South Africa may not be generalisable to other settings with different referral pathways, resource levels, and patient populations.

Unmeasured confounders: Despite multivariable adjustment, we cannot exclude residual confounding by unmeasured variables such as socioeconomic status, time to first debridement, nutritional status, or quality of care at referring hospitals.

Sample size: The small sample size limits statistical power to detect clinically important differences and precludes subgroup analyses (e.g., by mechanism of injury or specific delay duration).

### 4.7. Recommendations

Based on our findings and the significant limitations encountered, we make the following recommendations:

Clinical practice: The finding that early admission of the most severely injured patients is occurring suggests that current triage systems are functioning appropriately. However, the high rate of surgical intervention in delayed admissions warrants attention. Upskilling regional centres in early burn management, including escharotomy and appropriate fluid resuscitation, could mitigate the need for more extensive surgery later. Decentralising burn care where appropriate, with strong telemedicine support from the tertiary centre, could reduce the burden on the central unit and minimise waiting times for admission. Continuing medical education for emergency physicians on accurate TBSA estimation and early recognition of inhalational injury remains essential.

Research: The most critical recommendation is the urgent need for prospective data collection in real time. Developing standardised burn admission sheets, including Lund and Browder charts, would ensure that all necessary details are captured. Implementation of an electronic registry (e.g., REDCap database) for all burn centre admissions would facilitate complete data capture, enable audit and quality improvement, and allow for more robust research. A national burn registry for South Africa would enable multi-centre studies with adequate power to answer questions about optimal transfer timing and identify best practices across different settings.

Quality improvement: Regular audits of burn centre admissions and outcomes should be institutionalised. Specific attention should be paid to reasons for delayed admission, which were poorly documented in our cohort. Understanding these reasons (ICU bed availability, transfer logistics, COVID-19-related delays) is the first step towards addressing them. Infection control protocols should be reviewed, particularly given the strong association between Gram-negative sepsis and mortality.

### 4.8. Clinical Implications

While our findings must be interpreted cautiously due to the study limitations, several clinical implications emerge. The absence of a demonstrable harmful effect of delayed admission in this cohort should not be interpreted as evidence that delays are acceptable. Rather, it suggests that, with adequate interim management at referring centres, the negative impacts of transfer delay may be mitigated. This underscores the importance of investing in education and resources at district and regional hospitals to ensure that patients receive appropriate care while awaiting transfer.

The high surgical rate in delayed admissions may indicate that some patients could benefit from earlier intervention. Exploring barriers to timely surgery—including theatre access, staffing, and logistical challenges—should be a priority for quality improvement initiatives.

The strong association between Gram-negative sepsis and mortality reinforces the need for rigorous infection control measures, including protocolised line care, early wound excision where appropriate, and rational antibiotic use. Patients with large burns, flame burns, and delayed presentation should be considered high-risk for infectious complications and monitored accordingly.

Finally, the clustering of deaths in 2020 highlights the vulnerability of burn care systems to external shocks such as pandemics. Developing robust contingency plans for maintaining burn services during healthcare crises is essential. A further look at this population or time frame is warranted and planned by the authors.

## 5. Conclusions

In this single-centre retrospective cross-sectional study from a resource-constrained burn unit in Johannesburg, South Africa, delayed admission (>24 h) was not associated with increased length of stay, mortality, or sepsis. Early admission was independently associated with longer hospital stays, likely reflecting the rapid transfer of more severely injured patients requiring prolonged care. The delayed admission group required significantly more surgical interventions, suggesting possible progression of burn wound depth during the waiting period.

However, these findings must be considered preliminary due to critical limitations, including a small sample size representing only 8.7% of the total eligible population, a high risk of selection bias, and the inherent limitations of retrospective data collection. The lack of association between delayed admission and adverse outcomes may represent Type II error rather than true equivalence.

This study highlights the urgent need for prospective data collection and for establishing a national burn registry in South Africa. Only with robust, complete data can we adequately answer questions about optimal transfer timing, identify modifiable factors influencing outcomes, and implement effective quality improvement strategies in resource-limited settings. Burn care is costly, and accurate data are essential for appropriate resource allocation and for developing context-appropriate guidelines that reflect the realities of burn care in developing nations.

## Figures and Tables

**Table 1:** Descriptive statistics representing the demographic and clinical characteristics of patients admitted to the adult burn unit at CHBAH from 1 January 2018 to 31 December 2022.

Characteristic	Overall n = 123	Early (≤1 day) n = 71	Late (>1 day) n = 52	*p*-Value ^1^
Length of stay (days), median (IQR)	14 (8–25)	14 (9–24)	15 (7–27)	0.74
Sex, n (%)				0.094
Female	44 (36)	21 (30)	23 (44)	
Male	79 (64)	50 (70)	29 (56)	
Age (years), median (IQR)	34 (26–42)	32 (25–40)	36 (27–44)	0.26
TBSA (%), median (IQR)	17 (10–27)	16 (10–25)	18 (13–29)	0.62
Inhalation injury, n (%)	23 (19)	18 (25)	5 (9.6)	0.027
Baux score, median (IQR)	55 (44–74)	55 (44–77)	60 (45–73)	0.87
Reason for delayed admission, n (%)				0.019
Awaiting COVID-19 result	22 (18)	18 (25)	4 (7.7)	
Awaiting ICU bed	19 (15)	7 (9.9)	12 (23)	
Delayed presentation	5 (4.1)	2 (2.8)	3 (5.8)	
Not stated	77 (63)	44 (62)	33 (63)	
Sepsis, n (%)	33 (27)	19 (27)	14 (27)	0.98
Had any surgery, n (%)	41 (33)	15 (21)	26 (50)	<0.001
Has any identified organism, n (%)	24 (20)	13 (18)	11 (21)	0.69
Died, n (%)				0.34
Died	16 (13)	11 (15)	5 (9.6)	
Alive	107 (87)	60 (85)	47 (90)	
Year of admission, n (%)				0.071
2018	6 (4.9)	5 (7.0)	1 (1.9)	
2019	16 (13)	9 (13)	7 (13)	
2020	34 (28)	25 (35)	9 (17)	
2021	57 (46)	26 (37)	31 (60)	
2022	10 (8.1)	6 (8.5)	4 (7.7)	
Comorbidities				
Diabetes mellitus, n (%)	6 (4.9)	3 (4.2)	3 (5.8)	0.70
Hypertension, n (%)	12 (9.8)	6 (8.5)	6 (12)	0.57
Epilepsy, n (%)	5 (4.1)	2 (2.8)	3 (5.8)	0.65
Right ventricular dysfunction, n (%)	22 (18)	10 (14)	12 (23)	0.20
Mechanism of burn				
Chemical burn, n (%)	3 (2.4)	1 (1.4)	2 (3.8)	0.57
Electric burn, n (%)	21 (17)	18 (25)	3 (5.8)	0.004
Flame burn, n (%)	58 (47)	34 (48)	24 (46)	0.85
Hot water burn, n (%)	36 (29)	16 (23)	20 (38)	0.055
Steven Johnson Syndrome, n (%)	2 (1.6)	0 (0)	2 (3.8)	0.18
Parasuicide, n (%)	2 (1.6)	0 (0)	2 (3.8)	0.18

^1^ Wilcoxon rank sum test; Pearson’s Chi-squared test; Fisher’s exact test.

**Table 2:** Univariate and multivariate regression of factors associated with length of stay.

	Univariate			Multivariable	
Characteristic	N	Beta (95% CI)	*p*-Value	Beta (95% CI)	*p*-Value
Age (years)	123	0.20 (0.04 to 0.35)	0.017	0.36 (0.18 to 0.54)	<0.001
TBSA (%)	123	0.13 (−0.06 to 0.33)	0.17	0.26 (0.00 to 0.53)	0.054
Inhalation injury	123	5.5 (−0.77 to 12)	0.085	0.98 (−5.4 to 7.3)	0.76
Baux Score	123	0.15 (0.05 to 0.26)	0.005		
Early admission	123				
Early (≤1 day)		—		—	
Late (>1 day)		0.27 (−4.7 to 5.2)	0.92	−5.8 (−11 to −0.80)	0.024
Sepsis	123	9.2 (3.9 to 14)	<0.001	1.1 (−5.6 to 7.8)	0.75
Died	123	−5.1 (−12 to 2.2)	0.17	−21 (−31 to −11)	<0.001

Abbreviations: CI = Confidence interval.

**Table 3:** Table of descriptive statistics by death.

Characteristic	Overall n = 123	Died n = 16	Alive n = 107	*p*-Value ^1^
Length of stay(IQR)	14(8–25)	10(9–24)	15(9–26)	0.12
Age (years), median (IQR)	34 (26–42)	45 (34–69)	33 (25–39)	0.004
TBSA (%), median (IQR)	17 (10–27)	37 (34–50)	15 (10–22)	<0.001
Inhalational injury, n (%)	23 (19)	4 (25)	19 (18)	0.50
Baux Score, median (IQR)	55 (44–74)	88 (81–101)	53 (42–69)	<0.001
Early admission, n (%)				0.34
Early (≤1 day)	71 (58)	11 (69)	60 (56)	
Late (1 day)	52 (42)	5 (31)	47 (44)	
Sepsis, n (%)	33 (27)	4 (25)	29 (27)	>0.99

^1^ Wilcoxon rank sum test; Pearson’s Chi-squared test; Fisher’s exact test.

## Data Availability

The raw data supporting the conclusions of this article will be made available by the authors on request.
